# Inflammation response criteria for rheumatoid arthritis based on the two-component disease activity score

**DOI:** 10.1136/rmdopen-2025-006631

**Published:** 2026-03-09

**Authors:** Michael Stadler, Felice Rivellese, Darren Plant, Nisha Nair, Kimme L Hyrich, John Isaac, Ann Morgan, Anthony G Wilson, Suzanne M M Verstappen, Myles J Lewis, John Bowes, Costantino Pitzalis, Anne Barton

**Affiliations:** 1 Centre for Genetics and Genomics Versus Arthritis, The University of Manchester, Manchester, UK; 2 NIHR Manchester Biomedical Research Centre, Manchester University NHS Foundation Trust, Manchester, UK; 3 Centre for Experimental Medicine and Rheumatology, Queen Mary University of London, London, UK; 4 NIHR Barts Biomedical Research Centre, Barts Health NHS Trust, London, UK; 5 Centre for Epidemiology Versus Arthritis, The University of Manchester, Manchester, UK; 6 Translational and Clinical Research Institute, Newcastle University, Newcastle upon Tyne, UK; 7 NIHR Newcastle Biomedical Research Centre, Newcastle upon Tyne Hospitals NHS Foundation Trust, Newcastle upon Tyne, UK; 8 School of Medicine, University of Leeds, Leeds, UK; 9 NIHR Leeds Biomedical Research Centre, Leeds Teaching Hospitals NHS Trust, Leeds, UK; 10 UCD School of Medicine and Medical Science, Conway Institute, University College Dublin, Dublin, Ireland

**Keywords:** rheumatoid arthritis, synovitis, DMARD

## Abstract

**Objectives:**

To develop response criteria for rheumatoid arthritis (RA) using the two-component Disease Activity Score in 28 joints (2C-DAS28).

**Methods:**

Data were available for three stages of RA treatment progression, determined by the disease-modifying antirheumatic drug prescribed: early (on methotrexate; n=1051), established (on tumour necrosis factor inhibitors; n=989) and late RA (on second-line or later therapy; n=301). Inflammation response was defined as achieving remission or a clinically meaningful reduction in disease activity after 3 months of treatment. Corresponding 2C-DAS28 thresholds were determined using receiver operating characteristic analysis and Youden’s J, based on Boolean V.2.0 remission and clinical disease activity index response. The correlation of the proposed criteria with synovial thickness (ST) and power Doppler (PD) was assessed in an independent cohort (n=161) and compared with conventional response criteria. Finally, 6-month disease activity was compared between 3-month 2C-DAS28 responders and non-responders.

**Results:**

Thresholds to define inflammation response were 2C-DAS28<1.8 or a decrease in 2C-DAS28>1.7. In the validation cohort, 3-month 2C-DAS28 response was significantly correlated with lower ST (r=−0.25 (95% CI −0.47 to −0.04), p=0.037) and PD scores (r=−0.28 (95% CI −0.52 to −0.04), p=0.042). By contrast, conventional response criteria showed no significant correlation with synovitis scores. In the discovery cohorts, 3-month 2C-DAS28 responders retained lower disease activity at 6 months than non-responders.

**Conclusion:**

2C-DAS28 response correlates significantly with ultrasound-detected synovitis and is associated with improved clinical outcomes. This may support identification of biomarkers of treatment efficacy and clinical stratification, to identify patients with ongoing inflammation and those in whom disease activity is driven by non-inflammatory features.

**WHAT IS ALREADY KNOWN ON THIS TOPIC**

- A revised two-component Disease Activity Score in 28 joints (2C-DAS28) shows stronger correlation with ultrasound-assessed synovitis and joint erosions than conventional scores of clinical rheumatoid arthritis disease activity.

**WHAT THIS STUDY ADDS**

- This study introduces and validates response criteria based on the 2C-DAS28, and compares their performance with conventional response criteria, using ultrasound-assessed synovitis as the outcome measure.

**HOW THIS STUDY MIGHT AFFECT RESEARCH, PRACTICE OR POLICY**

- 2C-DAS28 response correlates more strongly with ultrasound-detected synovitis than conventional response criteria and could help studies aiming to identify reliable biomarkers of treatment response.
- If 2C-DAS28 scoring was used in combination with established scores, it could help to identify those patients whose disease activity is driven by non-inflammatory features that may not respond to treatment escalation; future prospective studies are required to investigate this.

## Introduction

 Rheumatoid arthritis (RA) is a systemic autoimmune disease characterised by synovial joint inflammation. Left untreated, this inflammation leads to irreversible joint damage and more severe, harder-to-treat disease; early successful treatment is therefore vital to improve long-term outcomes and quality of life.^1^ Thanks to advances in therapy, there are now a number of conventional and targeted disease-modifying antirheumatic drugs (DMARDs) licensed for use in RA, but RA is a highly heterogeneous disease,^2^ and the efficacy of individual agents varies from patient to patient.^3^ Identifying factors that may influence or predict response to treatment is a priority, to inform clinical decision-making and maximise the utility of available treatment options.^4^ Results so far, however, have been mixed, potentially because there is currently no agreed gold standard to measure RA-specific inflammation and disease progression that applies to all patients, and definitions of treatment response therefore differ across studies.^5^ This has motivated the development of a new inflammation-focused composite score of clinical RA disease activity (two-component Disease Activity Score in 28 joints (2C-DAS28)).^6^

In developing the 2C-DAS28, Hensor *et al*^6^ sought to reweigh the original four-component DAS28 (4C-DAS28) to maximise association with ultrasound-assessed synovitis. Testing the individual components of the 4C-DAS28, they found that only the swollen joint count (SJC28) and C reactive protein levels (CRP) were independently associated with ultrasound-assessed synovitis, while the tender joint count (TJC28) and the patient’s own global health assessment (PatGA) were not. In an independent validation cohort, the resulting 2C-DAS28 score showed stronger association with ultrasound-assessed synovitis and bone erosions than other conventional composite scores. We hypothesise that the 2C-DAS28 may more accurately determine whether treatment is adequately suppressing joint inflammation, but thresholds to assess treatment response have yet to be defined.

The TJC28 and PatGA are widely used components across conventional composite scores, but they may be influenced by non-inflammatory comorbidities^7 8^ and are not always associated with synovitis.^9^ This confounds results when assessing the efficacy of DMARD treatment, as demonstrated by multiple studies that found that some patients considered to be in clinical remission by conventional composite scores still have residual synovitis detectable with imaging techniques and are at increased risk of disease progression.^10^ Additionally, composite scores were originally introduced to be used in clinical trials, but systematic differences between patients in trial and registry populations have been documented, potentially exacerbating this confounding effect outside the stringent conditions of trials.^11^ Lastly, conventional treatment response criteria were designed in line with current treat-to-target goals by 6 months of treatment,^12^ but the 3-month timepoint is clinically critical, as it offers an opportunity to potentially adjust treatment in patients with inadequate response so far.^13^ Therefore, we aimed to develop and validate biological response thresholds based on the 2C-DAS28, to identify patients in whom inflammation was adequately controlled after 3 months of treatment and those with ongoing active synovitis.

## Methods

### Data sets

To explore biological response thresholds based on the 2C-DAS28, three cohorts were accessible, with clinical data available at baseline and at 3 months after treatment initiation. The three cohorts represent three distinct stages of RA treatment progression, determined based on the type of DMARD the patients were receiving. In the UK, treatment typically starts with conventional DMARDs, before progressing to tumour necrosis factor inhibitors (TNFi) and then other targeted therapies where necessary. Accordingly, the early RA cohort consists of patients from the Rheumatoid Arthritis Medication Study, a UK multicentre study that collects data on biologic-naive patients receiving methotrexate treatment for the first time.^14^ Data for the later treatment stages came from the Biologics in Rheumatoid Arthritis Genetics and Genomics Study Syndicate (BRAGGSS), which recruits patients starting biologic or targeted synthetic therapy and follows them prospectively.^15^ For the established RA cohort, we identified patients from BRAGGSS who were on their first course of TNFi. For the late RA cohort, we identified patients from BRAGGSS who had not responded to their initial biologic and were now starting a non-TNFi treatment. Lastly, the thresholds developed in the above cohorts were tested in an independent cohort of patients from the ‘Response, Relapse and Resistance to Rituximab in Rheumatoid Arthritis’ (R4RA) study receiving rituximab or tocilizumab.^16 17^ Validation data were available at 0 and 16 weeks postinitiation of therapy and additionally included synovial thickness (ST) and power Doppler (PD) scores as measures of ultrasound-detected synovitis, capturing structural progression and active inflammation, respectively.^18^ Ultrasound assessments were performed following the EULAR-Outcome Measures in Rheumatology (OMERACT) standardised protocol^19^: bilateral first to fifth metacarpophalangeal (MCP) joints and the wrists were scanned in the longitudinal dorsal plane with joints in a neutral position. This 12-joint reduced set was selected as it has been shown to be highly representative of more extensive joint counts, while maintaining high sensitivity to change in response to treatment.^20^ ST and PD were graded individually by a single scorer blind to treatment and timepoint, using a semi-quantitative scale (0–3) according to the EULAR-OMERACT consensus definitions.^18^ Representative images illustrating the grading criteria in the MCP joints, for both ST and PD, are provided in online supplemental figure S1. The individual joint scores were then summed to calculate the overall 12-max ST and PD scores (0–36 each) used for validation.

### Inflammation response criteria

Inflammation response was determined based on the 2C-DAS28, which combines SJC28 and CRP (mg/L)^6^: *2C-DAS28=√SJC28+0.6 ln(CRP+1*). Current EULAR recommendations suggest that patients should reach remission after 6 months and achieve at least a 50% decrease in disease activity by 3 months.^21^ These recommendations are based on conventional composite scores, which incorporate additional aspects of RA disease activity, not covered by the inflammation-focused 2C-DAS28. To compensate and prevent spurious results, more stringent thresholds than the recommended 50% were explored. Additionally, to maximise statistical power, 2C-DAS28 response was based on total change in score (Δ2C-DAS28), instead of percentage change^22^: Δ*2C-DAS28=2C-DAS28_BL_−2C-DAS28_FU_*.

Three-month 2C-DAS8 response was defined as reaching remission (target criterion) or achieving a clinically meaningful decrease in disease activity (improvement criterion). Corresponding 2C-DAS28 thresholds were determined based on conventional outcomes. 2C-DAS28 remission was defined independently of other composite scores, based on the Boolean V.2.0 remission criterion^23^: *TJC28≤1 and SJC28≤1 and CRP≤10 mg/L and PatGA≤20 mm*. The threshold for clinically meaningful decrease in 2C-DAS28 was determined based on response thresholds for the clinical disease activity index (CDAI).^24^ The CDAI was favoured over other outcomes because it has shown better alignment with radiographic outcomes.^25^ Additionally, the CDAI is purely clinical and does not weigh the subcomponent, making it highly general and widely applicable. We explored the views of patient partners (n=7) in the interpretation and use of outcome measures as part of a patient and public involvement and engagement event.

### Statistical analysis

Patients were included in this analysis if they had complete data to calculate the 2C-DAS28 and the CDAI at baseline and 3-month follow-up. 2C-DAS28 remission and improvement thresholds to define inflammation response were determined via receiver operating characteristic (ROC) analysis using conventional outcomes as anchors. Thresholds were assessed based on their discriminant validity using the true positive rate (sensitivity), the true negative rate (specificity) and the area under the ROC curve (AUC=[sensitivity+specificity]/2), and the ideal thresholds were chosen to maximise the sensitivity/specificity trade-off, using Youden’s J (sensitivity+specificity−1).^26^

Assuming baseline Boolean V.2.0 remission as ground truth, a ROC curve was constructed for the early RA cohort, by calculating the sensitivity and specificity of different 2C-DAS28 thresholds, varying in 0.1 increments (2C-DAS28<t), and the threshold with the highest J was chosen to define 2C-DAS28 remission. To define clinically meaningful improvement in 2C-DAS28, thresholds for minor, moderate and major decreases were determined assuming corresponding levels of percentage decrease in CDAI (ΔCDAI_%_>50%/70%/85%) as the ground truth. For each level of decrease, ROC curves for 2C-DAS28 decrease thresholds, varying in 0.1 decrements (Δ2C-DAS28>t), were constructed in each discovery cohort. The resulting ROC curves for every level were then pooled by calculating the weighted average sensitivity and specificity at each point, before again using Youden’s J to identify the ideal threshold. The improvement threshold with the highest discriminant validity, best capturing overall patient improvement, was deemed as clinically meaningful decrease in inflammation alone and selected as the improvement criterion to define 2C-DAS28 response.

The proposed response criteria were validated in the independent R4RA cohort. The discriminant validity with their respective anchor outcome was assessed, and disease components were compared in cases where criteria disagreed to understand which factors were driving discordance. 2C-DAS28 remission and response were structurally validated, by assessing their correlation (r) with ultrasound-detected synovitis, as measured by ST and PD, using the point-biserial correlation coefficient.^27^ Similarly, the correlation with the pain DAS28 (DAS28-P), calculated as the fraction of the 4C-DAS28 explained by the TJC28 and PatGA,^28^ was assessed. The remission threshold was further validated for its ability to predict imaging remission (PD=0),^29^ and results were compared with conventional outcomes based on the CDAI and 4C-DAS28. The EULAR response criteria were originally developed for the erythrocyte sedimentation rate (ESR) 4C-DAS28 and were therefore adjusted for the CRP 4C-DAS28 to facilitate comparison with the 2C-DAS28. Good or moderate EULAR_ESR_ response is defined as reaching moderate disease activity (4C-DAS28_ESR_≤5.1) with at least a moderate decrease in 4C-DAS28_ESR_ (σ_ESR_=Δ4C-DAS28_ESR_>0.6) or showing a major decrease in 4C-DAS28_ESR_ (Δ4C-DAS28_ESR_>2σ_ESR_).^30^ The moderate disease threshold for the 4C-DAS28_CRP_ has previously been defined as 4C-DAS28_CRP_≤4.6^31^ and a corresponding moderate decrease threshold (σ_CRP_) was determined via ROC analysis and Youden’s J.

Lastly, the proposed 2C-DAS28 criteria were temporally validated in the discovery cohorts using a subset of patients with 6-month follow-up data available, to investigate the potential clinical implications of inflammatory response. Binary logistic regression was used to assess the impact of 3-month 2C-DAS28 response on the likelihood of reaching clinical remission at 6 months (DAS28_CRP_<2.4^32^), and overall disease activity at 6 months was compared between 3-month 2C-DAS28 responders and non-responders, using two-tailed Mann-Whitney U tests.

Statistical analysis was conducted in Python (V.3.5.1). Point-biserial correlation and the associated t-test p value were calculated using the *pointbiserialr* function from the *scipy* package (V.1.6.2). Logistic regression models were fitted using the *Logit* function from the *statsmodels* package (V.0.13.2). Mann-Whitney U tests were conducted using the *mannwhitneyu* function from the *scipy* package (V.1.6.2).

## Results

There were 1051 patients in the early RA cohort with complete data to calculate the 2C-DAS28 and CDAI at baseline and 3-month follow-up, 989 patients in the established RA cohort and 301 patients in the late RA cohort. The discovery cohorts were predominantly female and matched for age, but expectedly, patients in the later stages had longer disease duration (median 0.7 years in the early RA cohort, compared with 5 and 13 years in the established and late RA cohorts, respectively), as well as higher baseline disease activity (table 1). Patients in the established RA cohort received adalimumab, certolizumab, etanercept, golimumab or infliximab, and patients in the late RA cohort received abatacept, baricitinib, rituximab, tocilizumab or tofacitinib (online supplemental table S1). The rate of seropositivity was markedly lower in the early RA cohort (~63%) than in the later cohorts (>80%), in line with other early disease cohorts (48%–63%).^33^ Additionally, a sizeable proportion of patients (44%–63%) presented with normal CRP levels (<10 mg/L) at baseline, consistent with previous findings (44%–58%).^34^

**Table 1:** Baseline cohort characteristics for the three discovery cohorts

	Early RA (n=1051)	Established RA (n=989)	Late RA (n=301)
Demographics			
Age	59 (±13) (0.4%)	57.4 (±12.2) (0.2%)	57.5 (±12.1) (1%)
Female	66.5%	76.9%	78.7%
BMI	28.2 (±5.8) (23.6%)	28.8 (±6.9) (17.3%)	29.6 (±6.9) (17.6%)
Seropositive	62.7% (20.7%)	83.1% (10.7%)	89.8% (2.1%)
Disease duration	2.4 (±5.1) (0.4%)	8.9 (±9.7) (1.4%)	12.8 (±12.3) (2.7%)
Disease activity			
SJC28	6.3 (±5.8)	9.2 (±5.2)	7.5 (±5.1)
TJC28	7.7 (±7.5)	15 (±6.8)	14 (±7.1)
CRP (mg/L)	14.6 (±24.1)	18.2 (±23.4)	25.2 (±32.9)
Normal CRP	62.7%	50.2%	43.5%
PatGA (mm)	41.2 (±23.6)	72.6 (±18.6)	74.0 (±17.5)
PhyGA (mm)	38.7 (±22.4)	67.1 (±17.3)	69.5 (±17.8)
2C-DAS28	3.4 (±1.5)	4.3 (±1.1)	4.1 (±1.3)
CDAI	21.9 (±14.3)	38.1 (±10.7)	35.9 (±11.8)
4C-DAS28	4.2 (±1.3)	5.7 (±0.8)	5.7 (±1)

Continuous factors are presented as mean±SD, and categorical factors as %-yes, relative to non-missing data. Percentages in brackets indicate the rate of missing data.

Disease duration: years since RA diagnosis. Normal CRP: CRP<10 mg/L. Seropositive: patient tested positive for anti-CCP or RF antibodies.

BMI, body mass index; CCP, cyclic citrullinated peptide; CDAI, clinical disease activity index; 2C-DAS28, two-component Disease Activity Score in 28 joints; 4C-DAS28, four-component DAS28 calculated from CRP; CRP, C reactive protein; PatGA, patient global health assessment; PhyGA, physician global health assessment; RA, rheumatoid arthritis; RF, rheumatoid factor; SJC28, swollen joint count; TJC28, tender joint count.

### Inflammation response criteria

The 2C-DAS28 remission threshold was determined using Boolean V.2.0 remission as an anchor. 46 patients (4.4%) in the early RA cohort were in baseline Boolean V.2.0 remission, and there were no patients in baseline Boolean V.2.0 remission in the later stage cohorts. Based on ROC analysis and Youden’s J, the 2C-DAS28 remission threshold was found to be 2C-DAS28<1.8 (online supplemental figure S2), and 168 (16%) patients in the early RA cohort were in baseline 2C-DAS28 remission (AUC=0.89; 95% CI 0.83 to 0.96; sensitivity=0.91; specificity=0.87).

**Figure 1:**
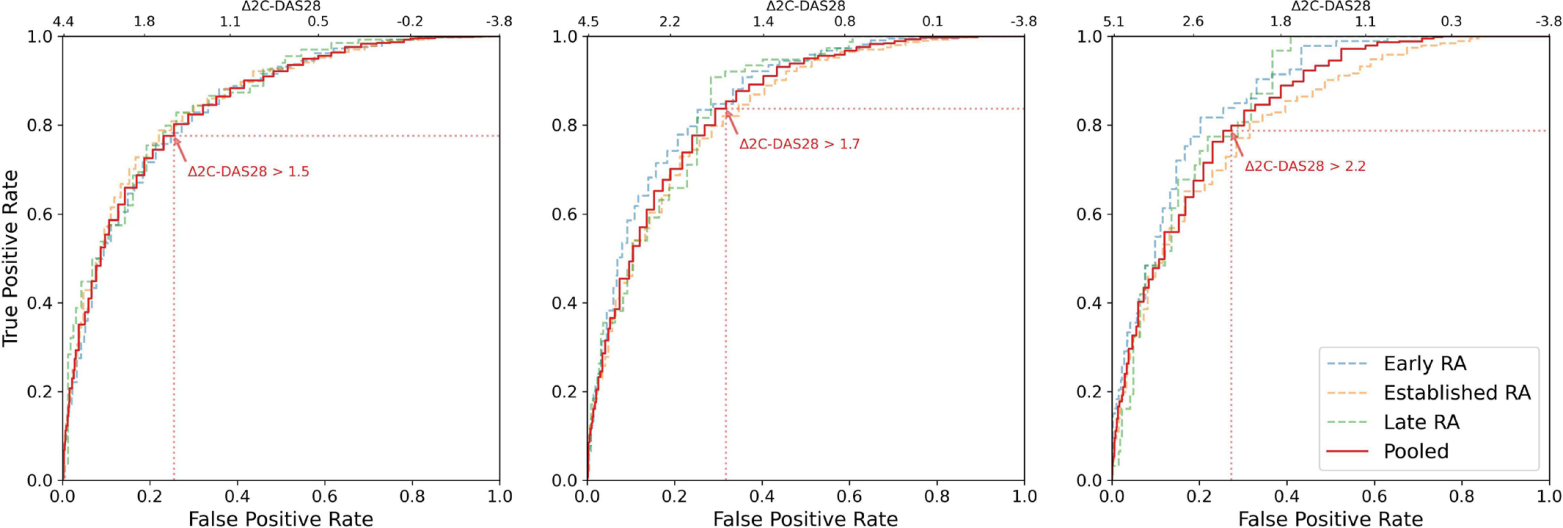
ROC curves for thresholds of minor (left), moderate (middle) and major (right) decrease in 2C-DAS28, based on corresponding thresholds of percentage decrease in CDAI (ΔCDAI_%_>50%/70%/85%). The individual curves were pooled using weighted averaging, and Youden’s J was used to identify the ideal thresholds (highlighted). Three ROC curves for 2C-DAS28 thresholds corresponding to minor, moderate and major decrease in CDAI, with ideal pooled thresholds, which were found to be 2C-DAS28>1.5, 2C-DAS28>1.7 and 2C-DAS28>2.2. 2C-DAS28, two-component Disease Activity Score in 28 joints; CDAI, clinical disease activity index; RA, rheumatoid arthritis; ROC, receiver operating characteristic.

Thresholds for decrease in 2C-DAS28 (Δ2C-DAS28) were determined using minor, moderate and major CDAI response (ΔCDAI_%_>50%/70%/85%) as anchors. Particularly in the early RA cohort, there were a high number of patients with very low disease activity at baseline, skewing the Δ2C-DAS28 distribution. Patients without active disease at baseline (2C-DAS28<1.8) were therefore excluded for this part of the analysis. Based on ROC analysis and Youden’s J, the pooled threshold for minor decrease in 2C-DAS28 was Δ2C-DAS28>1.5, >1.7 for moderate decrease in 2C-DAS28 and >2.2 for major decrease in 2C-DAS28 (figure 1). The three levels had consistent overall discriminant validity, but the moderate decrease threshold had the overall highest pooled sensitivity (online supplemental table S2). CDAI response additionally requires improvement in disease aspects not considered by the 2C-DAS28, and patients who have improved according to the CDAI should, on average, therefore also be expected to have improved according to the 2C-DAS28. Agreement on positives (sensitivity) was therefore deemed more important than agreement on negatives (specificity), and the moderate decrease threshold was taken forward to define inflammation response.

Inflammation response was thus defined as reaching 2C-DAS28 remission or achieving a moderate decrease in 2C-DAS28 (2C-DAS28<1.8 or Δ2C-DAS28>1.7). Across the discovery cohorts, there were 1278 (55%) 2C-DAS28 responders after 3 months, compared with 702 (30%) CDAI70 responders (pooled AUC=0.75 (95% CI 0.73 to 0.77); sensitivity=0.9; specificity=0.6). An Excel calculator to determine 2C-DAS28 response, based on baseline and follow-up SJC28 and CRP, is provided in online supplemental file 2.

### EULAR response for the 4C-DAS28_CRP_

The EULAR response criteria for the 4C-DAS28 were originally developed for the ESR 4C-DAS28^30^ and were therefore adjusted for the CRP 4C-DAS28. Using a subset of patients with additional data to calculate 4C-DAS28_ESR_ and 4C-DAS28_CRP_ at 6 months (n=1157), the threshold for moderate decrease in 4C-DAS28_CRP_ (σ_CRP_) was found to be Δ4C-DAS28_CRP_>0.8 (AUC=0.92 (95% CI 0.9 to 0.93); online supplemental figure S3). Good or moderate EULAR response based on the 4C-DAS28_CRP_ was thus defined as: (4C-DAS28_CRP_≤4.6 and Δ4C-DAS28_CRP_>0.8) or Δ4C-DAS28_CRP_>1.6, and the revised EULAR_CRP_ criteria had good discriminant validity with EULAR_ESR_ (AUC=0.9 (95% CI 0.88 to 0.92); sensitivity=0.86; specificity=0.93).

### Independent validation

There were 161 patients in the independent R4RA cohort with complete data to calculate the 2C-DAS28 and CDAI at baseline and 3-month follow-up. The validation cohort was representative of the discovery cohorts,^16^ with higher average disease duration and disease activity than the established RA cohort, but lower than the late RA cohort. ST and PD scores at baseline were available for 83 and 61 patients and at follow-up for 68 and 55 patients, respectively. At baseline, all composite scores were correlated with ST, but SJC28 and CRP were the only components individually associated with ST, while none of the tested outcomes were correlated with baseline PD (table 2). At follow-up, SJC28 and the 2C-DAS28 were correlated with ST, and SJC28 was additionally correlated with PD, where the 2C-DAS28 only showed hints of significance (online supplemental table S3).

**Table 2:** Correlation of clinical disease activity with ultrasound-assessed synovitis at baseline

	ST		PD	
	r (95% CI)	P value	r (95% CI)	P value
SJC28	**0.41 (0.21 to 0.57**)	**<0.01**	0.17 (−0.08 to 0.41)	0.2
TJC28	0.09 (−0.13 to 0.30)	0.4	−0.06 (−0.31 to 0.19)	0.6
CRP	**0.24 (0.02 to 0.43**)	**0.031**	0.23 (−0.02 to 0.46)	0.07*
PatGA	0.10 (−0.12 to 0.31)	0.4	0.11 (−0.14 to 0.35)	0.4
PhyGA	0.12 (−0.10 to 0.32)	0.3	0.11 (−0.14 to 0.36)	0.4
2C-DAS28	**0.38 (0.18 to 0.55**)	**<0.01**	0.16 (−0.10 to 0.39)	0.2
CDAI	**0.23 (0.01 to 0.42**)	**0.037**	0.07 (−0.19 to 0.32)	0.6
4C-DAS28	**0.27 (0.06 to 0.46**)	**0.012**	0.12 (−0.14 to 0.36)	0.4

Bold typeface indicates significance at p<0.05.

* Hint of significance.

CDAI, clinical disease activity index; 2C-DAS28, two-component Disease Activity Score in 28 joints; 4C-DAS28, four-component DAS28 calculated from CRP; CRP, C reactive protein; PatGA, patient global health assessment; PD, power Doppler; PhyGA, physician global health assessment; P value, correlation coefficient; r, point-biserial correlation; SJC28, swollen joint count; ST, synovial thickness; TJC28, tender joint count.

67 (42%) patients with R4RA achieved 2C-DAS28 remission after 16 weeks of treatment, compared with 17 patients (11%) who reached Boolean V.2.0 remission (AUC=0.79 (95% CI 0.66 to 0.92); sensitivity=0.94; specificity=0.65). Discordance between 2C-DAS28 and Boolean V.2.0 remission was predominantly due to 76% of patients in 2C-DAS28 remission not meeting Boolean V.2.0 remission, while, by contrast, only 1% of patients with active 2C-DAS28 disease were classified as being in Boolean V.2.0 remission. Discordant patients showed no significant difference in CRP levels but had higher TJC28, PatGA and PhyGA scores (online supplemental table S4). 2C-DAS28 remission was robustly correlated with lower ST (r=−0.37 (95% CI −0.57 to −0.17), p<0.01) and PD scores (r=−0.33 (−0.56 to −0.09), p=0.01). Conventional remission thresholds showed stronger correlation with pain and, except for CDAI, were also correlated with ST, but they were not correlated with PD (table 3). Furthermore, 2C-DAS28 remission had the highest overall positive and negative predictive value for predicting imaging remission (online supplemental table S5).

**Table 3:** Correlation of different remission thresholds with ultrasound-assessed synovitis and pain (mean±SD)

Remission	N_Yes_	Yes	No	r (95% CI**)**	P value
	**ST (n=68)**				
2C-DAS28	**22**	**12.2** (**±3.5**)	**17.7** (**±7.3**)	**−0.37 (−0.57 to −0.17**)	**<0.01**
Boolean V.2.0	**3**	**7.7** (**±2.1**)	**16.3** (**±6.8**)	**−0.26 (−0.48 to −0.04**)	**0.03**
CDAI	2	9 (±1)	16.1 (±6.8)	−0.18 (−0.40 to 0.05)	0.2
4C-DAS28_CRP_	**10**	**10.4** (**±3.3**)	**16.8** (**±6.9**)	**−0.33 (−0.54 to −0.12**)	**<0.01**
	**PD (n=55)**				
2C-DAS28	**17**	**2.4** (**±2.5**)	**5.5** (**±4.6**)	**−0.33 (−0.56 to −0.09**)	**0.01**
Boolean V.2.0	1	2 (±0)	4.6 (±4.4)	−0.08 (−0.34 to 0.18)	0.6
CDAI	1	2 (±0)	4.6 (±4.4)	−0.08 (−0.34 to 0.18)	0.6
4C-DAS28_CRP_	6	3 (±1.9)	4.7 (±4.5)	−0.12 (−0.38 to 0.14)	0.4
	**DAS28-P (n=161)**				
2C-DAS28	**67**	**0.4** (**±0.2**)	**0.5** (**±0.1**)	**−0.17 (−0.32 to −0.02**)	**0.03**
Boolean V.2.0	**17**	**0.1** (**±0.1**)	**0.5** (**±0.1**)	**−0.73 (−0.80 to −0.66**)	**<0.01**
CDAI	**14**	**0.1** (**±0.1**)	**0.5** (**±0.1**)	**−0.72 (−0.79 to −0.66**)	**<0.01**
4C-DAS28_CRP_	**33**	**0.2** (**±0.2**)	**0.5** (**±0.1**)	**−0.65 (−0.73 to −0.57**)	**<0.01**

Bold typeface indicates significance at p<0.05. P value for the correlation coefficient from a two-tailed t-test.

2C-DAS28 remission: 2C-DAS28<1.8; Boolean V.2.0: TJC28≤1 and SJC28≤1 and CRP≤10 mg/L and PatGA≤20 mm; CDAI≤2.8; 4C-DAS28_CRP_<2.4.

N_Yes_ indicates the number of patients in remission. Yes indicates patients in remission. No indicates patients not in remission.

CDAI, clinical disease activity index; 2C-DAS28, two-component Disease Activity Score in 28 joints; 4C-DAS28_CRP_, four-component DAS28 calculated from CRP; CRP, C reactive protein; DAS28-P, pain DAS28; PatGA, patient global health assessment; PD, power Doppler; r, point-biserial correlation; SJC28, swollen joint count; ST, synovial thickness; TJC28, tender joint count.

After 16 weeks of treatment, 96 (60%) patients were classified as 2C-DAS28 responders, compared with 36 (22%) CDAI70 responders (AUC=0.69 (95% CI 0.58 to 0.79); sensitivity=0.89; specificity=0.49). Consistent with 2C-DAS28 remission, discordance between the criteria was due to 67% of 2C-DAS28 responders not achieving CDAI70, while only 6% of 2C-DAS28 non-responders reached CDAI70. Discordant patients again showed no significant difference in CRP levels but had higher joint counts and global health assessments (table 4).

**Table 4:** Comparison of clinical disease activity (median±IQR) for discordant 2C-DAS28 responders

	CDAI70 response		
	Yes (n=67)	No (n=94)	P value
SJC	**0** (**±0.5**)	**1.5** (**±1.5**)	**<0.01**
TJC	**0.5** (**±1**)	**4** (**±4**)	**<0.01**
CRP (mg/L)	0 (±0.5)	1 (±2.5)	0.1
PatGA (mm)	**9** (**±9.8**)	**50** (**±13**)	**<0.01**
PhyGA (mm)	**10** (**±6.1**)	**30** (**±15.2**)	**<0.01**

Bold typeface indicates significance at p<0.05. P value from a two-tailed Mann-Whitney U test.

Yes indicates patients classified as 2C-DAS28 responders (2C-DAS28 response: 2C-DAS28<1.8 or Δ2C-DAS28>1.7) and CDAI70 responders (ΔCDAI_%_>70%). No indicates patients classified as 2C-DAS28, but not CDAI70 responders.

CDAI, clinical disease activity index; 2C-DAS28, two-component Disease Activity Score in 28 joints; CRP, C reactive protein; PatGA, patient global health assessment; PhyGA, physician global health assessment; SJC, swollen joint count; TJC, tender joint count.

**Figure 2:**
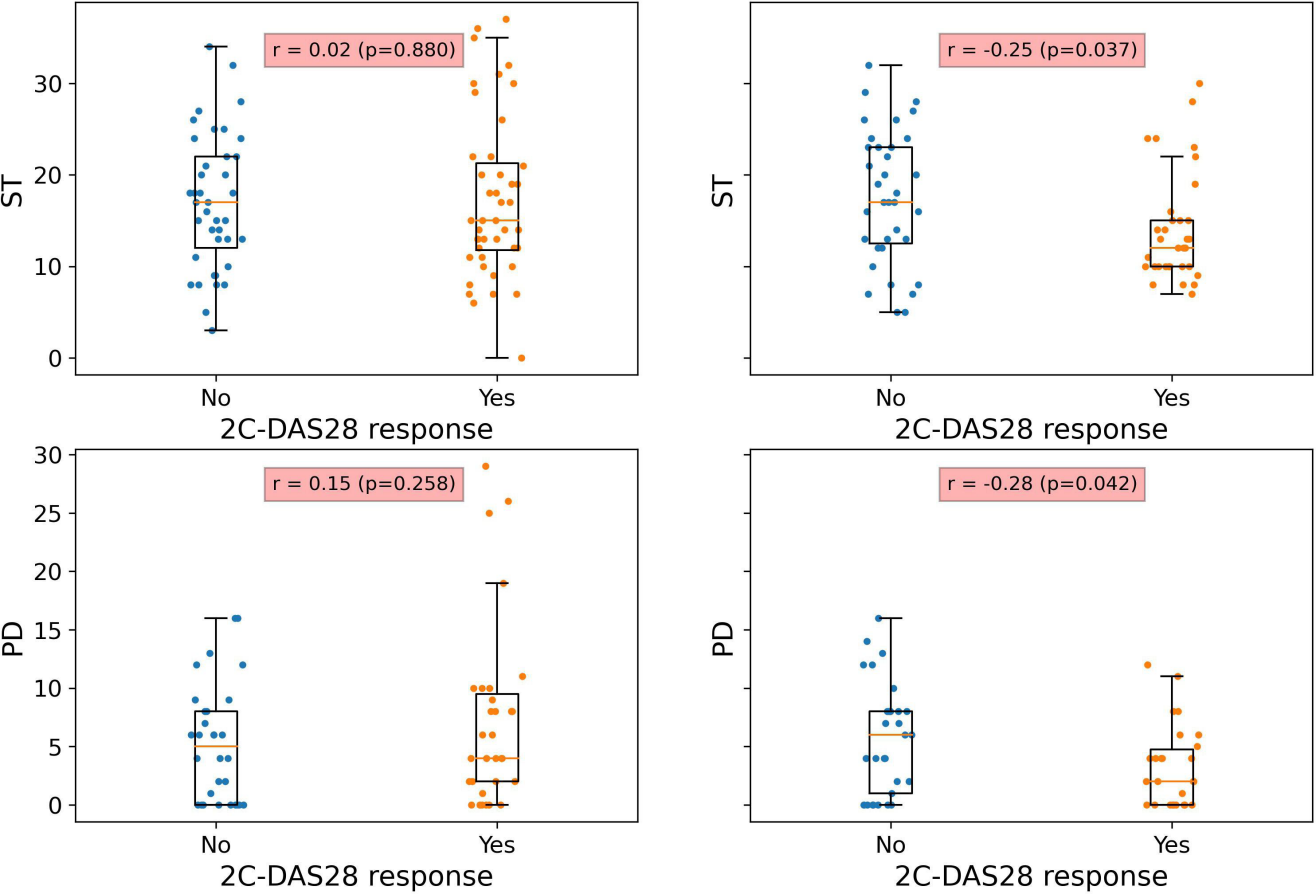
Comparison of pre-treatment (left column) and post-treatment (right column) ultrasound synovitis scores, between 2C-DAS28 responders and non-responders. Box plots comparing pre-treatment and post-treatment imaging synovitis scores between 2C-DAS28 responders and non-responders, showing that 2C-DAS28 responders improve significantly with respect to synovitis. 2C-DAS28 response, 2C-DAS28<1.8 or ∆2C-DAS28>1.7. P value for the correlation coefficient from a two-tailed t-test. 2C-DAS28, two-component Disease Activity Score in 28 joints; PD, power Doppler; r, point-biserial correlation; ST, synovial thickness.

2C-DAS28 response was correlated with significantly lower post-treatment ST (r=−0.25 (95% CI −0.47 to −0.04), p=0.037) and PD scores (r=−0.28 (95% CI −0.52 to −0.04), p=0.042), but there were no significant differences between responders and non-responders in pre-treatment synovitis scores (figure 2). Additionally, there were no significant differences for other clinical factors, including potential confounders such as body mass index (BMI)^35^ or normal CRP (online supplemental table S6). The baseline rate of normal CRP levels in the R4RA cohort (45%) was representative of the discovery cohorts, and there were no significant differences in rates of normal CRP between 2C-DAS28 responders and non-responders (p=0.8). Conventional response criteria, based on CDAI and 4C-DAS28, were not correlated with synovitis but showed stronger correlation with pain than 2C-DAS28 response (table 5).

**Table 5:** Correlation of different response criteria with ultrasound-assessed synovitis and pain (mean±SD)

Response	N_Yes_	Yes	No	r (95% CI)	P value
	**ST (n=68)**				
2C-DAS28	**33**	**14.1 (±5.9)**	**17.6 (±7.2)**	**−0.25 (−0.47 to −0.04)**	**0.037**
CDAI_70_	13	12.8 (±4.6)	16.6 (±7.1)	−0.22 (−0.44 to 0.01)	0.08
EULAR_CRP_	40	15.4 (±6.6)	16.5 (±7.2)	−0.08 (−0.32 to 0.16)	0.5
CDAI_3mo_	28	15.6 (±7.2)	16.1 (±6.6)	−0.04 (−0.28 to 0.2)	0.8
4C-DAS28_3mo_	16	13.4 (±6.1)	16.6 (±6.9)	−0.2 (−0.42 to 0.03)	0.1
	**PD (n=55)**				
2C-DAS28	**26**	**3.3 (±3.4)**	**5.7 (±4.7)**	**−0.28 (−0.52 to −0.04)**	**0.042**
CDAI_70_	8	3.5 (±2.4)	4.7 (±4.6)	−0.10 (−0.36 to 0.16)	0.5
EULAR_CRP_	31	4.0 (±4)	5.2 (±4.7)	−0.14 (−0.40 to 0.12)	0.3
CDAI_3mo_	20	4.4 (±4.1)	4.6 (±4.5)	−0.02 (−0.29 to 0.24)	0.9
4C-DAS28_3mo_	9	3.7 (±3.1)	4.7 (±4.5)	−0.09 (−0.35 to 0.17)	0.5
	**DAS28-P (n=161)**				
2C-DAS28	96	0.4 (±0.2)	0.5 (±0.1)	−0.10 (−0.26 to 0.05)	0.2
CDAI_70_	**36**	**0.2 (±0.2)**	**0.5 (±0.1)**	**−0.66 (−0.74 to −0.59)**	**<0.01**
EULAR_CRP_	**106**	**0.4 (±0.2)**	**0.5 (±0.1)**	**−0.34 (−0.47 to −0.21)**	**<0.01**
CDAI_3mo_	**80**	**0.4 (±0.2)**	**0.5 (±0.1)**	**−0.52 (−0.62 to −0.41)**	**<0.01**
4C-DAS28_3mo_	**43**	**0.3 (±0.2)**	**0.5 (±0.1)**	**−0.61 (−0.7 to −0.52)**	**<0.01**

2C-DAS28 response: 2C-DAS28<1.8 or ∆2C-DAS28>1.7; CDAI_70_: CDAI≤10 and ∆CDAI_%_>70%; EULAR_CRP_: 4C-DAS28_CRP_≤4.6 and Δ4C-DAS28_CRP_>0.8 or Δ4C-DAS28_CRP_>1.6; CDAI_3mo_: CDAI≤2.8 or ΔCDAI_%_>50%; 4C-DAS28_3mo_: 4C-DAS28_CRP_<2.4 or Δ4C-DAS28_CRP %_>50%.

Bold typeface indicates significance at p<0.05. P value for the correlation coefficient from a two-tailed t-test.

No indicates non-responders; yes indicates responders; N_Yes_ indicates the number of responders.

CDAI, clinical disease activity index; 2C-DAS28, two-component Disease Activity Score in 28 joints; 4C-DAS28, four-component DAS28; CRP, C reactive protein; DAS28-P, pain DAS28; PD, power Doppler; r, point-biserial correlation; ST, synovial thickness.

### Internal temporal validation

Across the discovery cohorts, data to additionally calculate the 4C-DAS28 at 6 months were available for 1758 patients. 987 patients (56.1%) were 3-month 2C-DAS28 responders, and 492 (28%) patients reached 4C-DAS28 remission at 6 months. Logistic regression showed that, compared with non-responders, 3-month 2C-DAS28 responders were more than three times as likely to reach 6-month 4C-DAS28 remission (pooled OR=3.41 (95% CI 1.64 to 7.09)). Three-month 2C-DAS28 response was additionally associated with overall lower disease activity at 6 months, and in the early and established RA cohort, it was also associated with lower impairment and pain (table 6).

**Table 6:** Comparison of clinical disease activity (median±IQR) after 6 months of treatment, between 3-month 2C-DAS28 responders and non-responders

	N	Yes	No	P value
	**4C-DAS28**			
Early RA	**886**	**2.7 (±0.9)**	**3.6 (±1)**	**<0.01**
Established RA	**669**	**2.8 (±0.9)**	**3.7 (±1)**	**<0.01**
Late RA	**203**	**3.4 (±1.1)**	**4.4 (±0.9)**	**<0.01**
	**CDAI**			
Early RA	**901**	**7.5 (±5.3)**	**15 (±8.4)**	**<0.01**
Established RA	**638**	**9 (±6)**	**15.5 (±9)**	**<0.01**
Late RA	**184**	**13 (±9.4)**	**21.5 (±7.9)**	**<0.01**
	**DAS28-P**			
Early RA	**886**	**0.4 (±0.2)**	**0.5 (±0.1)**	**<0.01**
Established RA	**669**	**0.4 (±0.1)**	**0.5 (±0.1)**	**<0.01**
Late RA	203	0.4 (±0.1)	0.5 (±0.1)	0.2
	**HAQ**			
Early RA	**784**	**0.6 (±0.6)**	**1 (±0.6)**	**<0.01**
Established RA	**559**	**1.1 (±0.7)**	**1.5 (±0.6)**	**<0.01**
Late RA	152	1.9 (±0.4)	1.9 (±0.5)	0.8

Bold typeface indicates significance at p<0.05. P value from a two-tailed Mann-Whitney U test.

N indicates the number of samples with non-missing data at 6-month follow-up. Yes indicates 2C-DAS28 responders after 3 months of treatment (2C-DAS28<1.8 or ∆2C-DAS28>1.7). No indicates 2C-DAS28 non-responders.

CDAI, clinical disease activity index; 2C-DAS28, two-component Disease Activity Score in 28 joints; 4C-DAS28, four-component DAS28; DAS28-P, pain DAS28; HAQ, Health Assessment Questionnaire; RA, rheumatoid arthritis.

### Patient involvement

In an informal half hour round table discussion, after being presented with a preliminary version of this work, patient representatives were invited to share their thoughts on outcome measures that omit patient-reported outcomes. The consensus of the patient representatives was that for them, it was important to know whether treatment is working or not and they had no concerns when considering, for example, the 2C-DAS28 when assessing response to treatment, if their general well-being, especially with respect to pain, was still considered in clinical management.

## Discussion

Here, we developed criteria to identify patients who, in response to treatment, markedly improved with respect to clinically detected inflammation, and those who did not, based on the recently proposed, inflammation-focused, 2C-DAS28,^6^ calculated from the SJC28 and CRP. Using three discovery cohorts of patients spanning three distinct stages of RA treatment progression, we determined thresholds of 2C-DAS28 remission and clinically meaningful decrease in 2C-DAS28, and patients who met either threshold (2C-DAS28<1.8 or Δ2C-DAS28>1.7) after 3 months of treatment were denoted as 2C-DAS28 responders. In a fourth, independent cohort, 3-month 2C-DAS28 response was robustly correlated with lower ST and PD ultrasound scores at follow-up. This indicates an improvement in structural progression and active joint inflammation, respectively,^18^ demonstrating that the proposed criteria correctly identify a subset of patients where treatment is working to suppress joint inflammation. Notably, there were no significant baseline differences in synovitis ultrasound scores or other clinical factors between 3-month 2C-DAS28 responders and non-responders to potentially explain this decrease, corroborating that this improvement is indeed due to treatment.

By comparison, no significant correlations between ultrasound-assessed synovitis and conventional response criteria, based on CDAI or 4C-DAS28, were detected. In this validation cohort, only SJC28 and CRP correlated with synovitis scores at baseline, but TJC28 and PatGA did not, consistent with previous results.^6^ As TJC28 and PatGA are included in the CDAI and the 4C-DAS28, this may explain why no correlation was detected. Importantly, conventional response criteria were developed for the 6-month timepoint, while we chose to classify inflammation response at the 3-month timepoint, as this offers opportunities to adjust treatment in patients where treatments are slow to be effective.^13^ It is possible that this difference in the timing at which response is classified may explain some of these findings. Similarly, the EULAR criteria for the 4C-DAS28 were originally developed for the ESR 4C-DAS28 and therefore had to be adjusted for comparison with the CRP-based 2C-DAS28, which may further contribute to these results. Notably, however, the revised CRP criteria had high agreement with their ESR counterpart, and there was no significant correlation with synovitis for the suggested EULAR 3-month 50% threshold^21^ either.

Overall, these results further corroborate that conventional composite scores and associated response criteria do not necessarily correlate with active joint inflammation early during treatment. Similarly, while 2C-DAS28 remission was associated with lower ST and PD scores, Boolean V.2.0 and 4C-DAS28 remission were only correlated with ST, and CDAI remission was not correlated with either. Concordantly, 2C-DAS28 remission was the best predictor of imaging remission (absence of PD signal^29^). Overall, our findings demonstrate that, as hypothesised, inflammation-focused outcome criteria correlate better with active joint inflammation than conventional scoring criteria. However, it is worth noting that this study was limited to commonly used composite measures of clinical characteristics. Disease activity and response to treatment can also be assessed directly via ultrasound or via an integrated approach, for a more biologically accurate estimate of active synovitis.^36^ However, despite growing evidence of ultrasound’s utility, uncertainty on which joints to scan to capture global RA disease activity and costs presents barriers for widespread adoption in research and clinical practice.^37^ Baker *et al*^38^ have previously proposed another inflammation-focused composite score of clinical RA disease activity, which may also hold promise to assess whether treatment is suppressing joint inflammation, although this score also incorporates the physician’s global health assessment, which is less readily accessible across research cohorts,^6^ and was not associated with ultrasound outcomes in this study.

Compared with these alternatives, 2C-DAS28 is widely applicable across existing data sets and consistent with current practice. 2C-DAS28 outcomes correlate with joint inflammation, plus the SJC28 and CRP show higher heritability than TJC28 and the PatGA.^39^ Together this means that the proposed criteria should improve power to detect reliable biomarkers of inflammation resolution in response to treatment. Notably, however, although statistically significant, the magnitude of the detected correlation for the proposed 2C-DAS28 criteria remained low (r~0.3).^40^ The reduced set of 12 joints assessed for ultrasound synovitis in this study compared with the 16 additional joints covered by the 2C-DAS28 (PIPs 1–5, shoulders, elbows, knees) and other confounding factors not captured by the 2C-DAS28 alone, such as RA disease heterogeneity^2^ or subclinical inflammation detected via ultrasound,^41^ may be limiting the strength of the correlation. Additionally, treatments like interleukin-6 inhibitors (eg, tocilizumab) can suppress CRP, a key component of the 2C-DAS28, independently of synovitis.^42^ This can lead to some patients being classified as responders, despite no decrease in active joint inflammation, which may negatively impact the detected correlation. CRP levels may also be elevated by non-synovial inflammation due to comorbidities,^43^ further confounding the correlation with active synovitis. It has therefore previously been suggested that single-outcome criteria, for example, using SJC28 alone, may be the most appropriate.^44^ Nonetheless, uncontrolled CRP has been associated with worse radiographic progression,^45^ and it remains part of current recommendations as an important biomarker for assessment.^12^ Additionally, in the cohorts in which the 2C-DAS28 was originally developed and validated, CRP remained significantly associated with ultrasound-assessed synovitis, independently of SJC28. Consistent with results of other inflammation-focused composite scores,^38^ this suggests that CRP may capture additional information about synovitis. Blood inflammation markers that better reflect RA-specific disease activity^46^ have high potential to further improve this correlation between clinical measures and synovial inflammation, but they remain an area for future research.

In the discovery cohorts, 3-month 2C-DAS28 response was additionally associated with lower disease activity at 6 months across all tested components, including non-inflammatory ones, and concordantly, 2C-DAS28 responders were significantly more likely than non-responders to reach the 6-month treat-to-target goal of clinical composite score remission. In the early and established RA cohorts, but not in the late RA cohort, 3-month 2C-DAS28 response was additionally associated with lower 6-month pain and impairment scores. A study in difficult-to-treat RA additionally found that high SJC28 and CRP, as well as persistent ultrasound-detected synovitis, are predictive of faster radiographic progression.^47^ Together, these findings show the benefit and importance of early inflammation control, highlighting the potential clinical utility of inflammation response criteria. When developing a revised outcome measure, it is important that it is not a duplication of existing criteria, and expectedly, the proposed 2C-DAS28 criteria show only moderate agreement with conventional outcomes. Most patients who satisfied conventional response criteria also reached 2C-DAS28 response, but a sizeable portion of 2C-DAS28 responders was not considered responders by conventional criteria. Similarly, only 1% of patients who achieved Boolean V.2.0 remission did not also achieve 2C-DAS28 remission, while a considerable number of patients who achieved 2C-DAS28 remission did not achieve Boolean V.2.0 remission. Importantly, however, for both remission and response, there were no significant differences in clinical inflammation for discordant samples. Instead, disagreement between the criteria was driven by TJC28 and PatGA. While these features are not associated with ultrasound-assessed synovitis, they are important measures of overall patient well-being, and particularly pain. Conventional outcomes correlated more strongly with pain than the proposed 2C-DAS28 thresholds, but it is recognised that pain may persist even if synovitis is alleviated, particularly in later stages of RA when joint damage may have already accumulated. Additionally, we have previously reported that psychological factors correlate with the PatGA.^48^ These factors may continue to impact well-being even if joint inflammation is effectively controlled by treatment, and failure to account for them may confound the clinical assessment of patients. A recent study found that 40% of patients classified as difficult-to-treat showed no objective signs of active inflammation, but instead had higher prevalence of BMI and fibromyalgia, affecting their overall well-being and potentially contributing to their classification as difficult-to-treat.^49^ In some cases, this may lead to a step-up in treatment intensity, with potentially more adverse events, despite current DMARD treatment effectively suppressing joint inflammation.

Importantly, while ultrasound holds promise to more accurately capture active synovitis, it is worth noting that clinical trials so far have not demonstrated a long-term benefit of targeting ultrasound remission over composite score remission,^50^ and not all ultrasound features are representative of active inflammation.^51^ At the same time, joint tenderness, not correlated with ultrasound-assessed synovitis directly, and therefore omitted from the 2C-DAS28, has been associated with subsequent radiographic progression at later follow-ups.^52^ RA is a complex heterogeneous disease with varying clinical manifestations, not all of which can be fully captured by ultrasound and markers of clinical inflammation alone. Inflammation response criteria should therefore not aim to replace but rather complement conventional outcomes to assess response to treatment. By assessing inflammatory response in combination with conventional outcomes, different subgroups of patients may be identified more readily. Patients in whom treatment has not been effective in suppressing inflammation may require more intensive treatment to achieve established treatment goals, whereas for patients in whom treatment is effective but pain and/or well-being does not improve, adjunctive support may be provided without necessarily requiring a change or step-up in DMARD therapy.

Strengths of the study include the use of the three discovery cohorts with over 2300 patients, spanning three different treatment stages of RA. The proposed criteria were additionally temporally validated at a later follow-up, and further structurally validated in a fourth independent cohort. However, a limitation of this study is the lack of synovitis scores for the discovery cohorts, which may otherwise have been used as independent anchors to define response instead of using derived outcomes. Another limitation was the modest size of the validation data set, only including patients at later treatment stages and only covering two DMARD agents, and the 2C-DAS28 response thresholds require validation across all treatment stages of RA and across a wider variety of available DMARD options. Particularly noteworthy is the lack of imaging validation for early RA, an important clinical subgroup that offers unique opportunity to prevent progression to more difficult-to-treat disease stages through early and effective treatment.^1^ While patients on first-line treatment formed a sizeable portion of the discovery cohorts (~45%), further external validation with radiographic outcomes is urgently needed. Similarly, temporal validation in this study was limited to one subsequent follow-up at 6 months, and further longitudinal validation studies are needed to fully assess the impact of 2C-DAS28 response on long-term clinical outcomes. Another important limitation is that the number of joints assessed by ultrasound in the independent validation cohort was limited, which prevented a more comprehensive analysis of global imaging detected synovitis. Inflammation in RA-affected joints beyond the scope of current composite scores (eg, ankles and feet)^53^ may contribute to these findings but could not be assessed with the available data. Further studies are warranted to investigate the relationship between 28-joint composite scores and inflammation in these other joints, overall and in response to treatment. Finally, focusing on 2C-DAS28 may neglect other important aspects of RA management that impact pain and a patient’s overall well-being, but integrating the 2C-DAS28 with existing assessments may provide important insights into specific aspects that require tailored interventions to improve the overall well-being. Notably, while the patient representatives involved as part of this study expressed no concerns with potentially incorporating the 2C-DAS28 in clinical practice, their views may not reflect the view of all patients, and patient engagement in this study was limited. Further formal investigations are required to explore this fully and should form part of any future 2C-DAS28 validation studies.

In summary, we propose criteria based on the new 2C-DAS28 score to identify patients who show marked improvement in clinical inflammation after 3 months of treatment and those who do not. We demonstrate that the proposed criteria correlate more strongly with active joint inflammation than conventional outcomes, which could aid research to identify biomarkers of targeted treatment response.^54^ Early inflammation control was additionally associated with overall improved clinical outcomes, further indicating that these criteria could complement conventional outcomes to inform the clinical management of patients.

## Supplementary material

10.1136/rmdopen-2025-006631 online supplemental file 1

10.1136/rmdopen-2025-006631 online supplemental file 2

## Data Availability

Data are available on reasonable request.
