# Supplementary material for: Inflammation response criteria for rheumatoid arthritis based on the two-component disease activity score
Source: RMD Open. 2026 Mar 9;12(1):e006631. doi: 10.1136/rmdopen-2025-006631 (PMC12983693; doi:10.1136/rmdopen-2025-006631)
Supplement: online supplemental file 1 [file rmdopen-12-1-s001.docx]

Supplementary material

**Supplementary Tables**

**Supplementary Table S1** - Distribution of different DMARDs in the established and late RA discovery cohorts

**Supplementary Table S2** - Pooled discriminant validity of 2C-DAS28 of minor, moderate, and major decrease in 2C-DAS28 compared to corresponding CDAI %-decrease thresholds

**Supplementary Table S3** - Correlation of clinical disease activity with ultrasound-assessed synovitis after treatment

**Supplementary Table S4** - Comparison of clinical disease activity for discordant patients in 2C-DAS28 remission

**Supplementary Table S5** - Validation of different remission criteria for predicting imaging remission

**Supplementary Table S6** - Comparison of baseline clinical characteristics between 2C-DAS28 responders and non-responders

**Supplementary Figures**

**Supplementary Figure S1** - Semi-quantitative grading of synovitis in the MCP joints according to the EULAR-OMERACT consensus definitions

**Supplementary Figure S2** - ROC curve for thresholds of 2C-DAS28 remission

**Supplementary Figure S3** - ROC curves for thresholds of moderate change in 4C-DAS28_CRP_

| **DMARD** | **Cohort** | **Type** | **N (%)** | |
| --- | --- | --- | --- | --- |
| Etanercept | Established | TNFi | 486 | (49.1%) |
| Adalimumab | Established | TNFi | 290 | (29.3%) |
| Certolizumab | Established | TNFi | 141 | (14.3%) |
| Golimumab | Established | TNFi | 46 | (4.7%) |
| Infliximab | Established | TNFi | 26 | (2.6%) |
| Rituximab | Late | non-TNFi | 151 | (50.2%) |
| Tocilizumab | Late | non-TNFi | 103 | (34.2%) |
| Abatacept | Late | non-TNFi | 38 | (12.6%) |
| Baricitinib | Late | non-TNFi | 7 | (2.3%) |
| Tofacit | Late | non-TNFi | 2 | (0.7%) |

Supplementary Table S1: Distribution of DMARDs in the established and late RA discovery cohorts.

|  | **N_CDAI_** | **N_Δ2C-DAS28_** | **Sens** | **Spec** | **AUC [95% CI]** |
| --- | --- | --- | --- | --- | --- |
| Minor decrease | 1124 (52%) | 1137 (53%) | 0.78 | 0.74 | 0.76 [0.74 0.78] |
| Moderate decrease | 689 (32%) | 1033 (48%) | 0.84 | 0.68 | 0.76 [0.74 0.78] |
| Major decrease | 316 (15%) | 745 (35%) | 0.79 | 0.73 | 0.76 [0.73 0.79] |

Supplementary Table S2: Pooled discriminant validity of minor, moderate, and major decrease in 2C-DAS28 compared to corresponding CDAI %-decrease thresholds (N_CDAI_: number of patients with minor/moderate/major %-decrease in CDAI [ΔCDAI_%_ > 50/70/85%]; N_Δ2C-DAS28_: number of patients with minor/moderate/major decrease in 2C-DAS28 [Δ2C-DAS28 > 1.5/1.7/2.2]; Sens: sensitivity; Spec: specificity; AUC: area under the ROC-curve).

|  | **ST** | | **PD** | |
| --- | --- | --- | --- | --- |
|  | ***r [95% CI]*** | ***p*** | ***r [95% CI]*** | ***p*** |
| SJC28 | **0.36 [0.13 0.55]** | **0.002** | **0.33 [0.07 0.55]** | **0.01** |
| TJC28 | 0.01 [-0.23 0.25] | 0.9 | 0.03 [-0.24 0.29] | 0.8 |
| CRP (mg/L) | 0.03 [-0.21 0.27] | 0.8 | -0.04 [-0.30 0.23] | 0.8 |
| PatGA (mm) | 0.10 [-0.14 0.33] | 0.4 | -0.04 [-0.30 0.23] | 0.8 |
| PhyGA (mm) | 0.05 [-0.19 0.29] | 0.7 | -0.02 [-0.28 0.25] | 0.9 |
| 2C-DAS28 | **0.33 [0.09 0.52]** | **p < 0.01** | 0.25 [-0.02 0.48] | 0.07** |
| CDAI | 0.18 [-0.06 0.40] | 0.2 | 0.11 [-0.16 0.36] | 0.5 |
| 4C-DAS28 | 0.16 [-0.09 0.38] | 0.2 | 0.06 [-0.20 0.32] | 0.6 |

*Supplementary Table S3: Correlation of clinical disease activity with ultrasound-assessed synovitis after treatment (ST: synovial thickness; PD: Power Doppler; SJC28: swollen joint count; TJC28: tender joint count; CRP: C-reactive protein levels; PatGA: patient global health assessment; PhyGA: physician global health assessment; 2C-DAS28: 2-component DAS28; CDAI: clinical disease activity index; 4C-DAS28: 4-component DAS28 calculated from CRP; r: point-biserial correlation; p: p-value for the correlation coefficient; bold typeface indicates significance at p < 0.05 [** hint of significance]).*

|  | **Boolean2.0 remission** | | |
| --- | --- | --- | --- |
|  | ***Yes (N = 67)*** | ***No (N = 94)*** | ***p*** |
| SJC | **0 (± 0)** | **0 (± 1)** | **0.02** |
| TJC | **0 (± 0)** | **3 (± 2.2)** | **p < 0.01** |
| CRP (mg/L) | 0 (± 0.5) | 0 (± 0.5) | 0.85 |
| PatGA (mm) | **3.5 (± 4)** | **41 (± 16.5)** | **p < 0.01** |
| PhyGA (mm) | **4.5 (± 5.2)** | **23 (± 11.2)** | **p < 0.01** |

Supplementary Table S4: Comparison of clinical disease activity (median ±IQR) for discordant patients in 2C-DAS28 remission (Yes: patients in 2C-DAS28 remission [2C-DAS28 < 1.8] and Boolean2.0 remission; No: patients in 2C-DAS28, but not Boolean2.0 remission).

| **Remission** | **TP** | **FP** | **TN** | **FN** | **Sens** | **Spec** | **PPV** | **NPV** |
| --- | --- | --- | --- | --- | --- | --- | --- | --- |
| 2C-DAS28 | 6 | 11 | 28 | 10 | 0.38 | 0.72 | 0.35 | 0.74 |
| Boolean2.0 | 0 | 1 | 38 | 16 | 0 | 0.97 | 0 | 0.70 |
| CDAI | 0 | 1 | 38 | 16 | 0 | 0.97 | 0 | 0.70 |
| 4C-DAS28_CRP_ | 1 | 5 | 34 | 15 | 0.06 | 0.87 | 0.17 | 0.69 |

Supplementary Table S5: Validation of different remission criteria for predicting imaging remission (2C-DAS28 remission: 2C-DAS28 < 1.8; Boolean2.0: TJC28 ≤ 1 and SJC28 ≤ 1 and CRP ≤ 10 mg/L and PatGA ≤ 20 mm; CDAI: CDAI ≤ 2.8; 4C-DAS28_CRP_: 4C-DAS28_CRP_ < 2.4; TP = true positives; FP = false positives; TN = true negatives; FN = false negatives; Sens: sensitivity; Spec: specificity; PPV = positive predictive value; NPV = negative predictive value).

|  | **2C-DAS28 response** | | |
| --- | --- | --- | --- |
|  | ***Yes*** | ***No*** | ***p*** |
| BMI | 26.1 (±3.9) | 27.5 (±3.9) | 0.2 |
| SJC28 | 5.5 (± 4) | 6 (± 3) | 0.6 |
| TJC28 | 10 (± 6) | 12 (± 5) | 0.2 |
| CRP (mg/L) | 10.5 (± 15.1) | 14 (± 7) | 0.6 |
| normal CRP | 46.9% | 58.5% | 0.8 |
| PatGA (mm) | 73.5 (± 19.2) | 71 (± 15.5) | 0.3 |
| PhyGA (mm) | 60 (± 14.6) | 64 (± 15) | 0.5 |

Supplementary Table S6: Comparison of baseline clinical characteristics between 2C-DAS28 responders and non-responders (Continuous factors are presented as median ± IQR, and categorical factors as %-yes; Yes: 2C-DAS28 responders [2C-DAS28 < 1.8 or ∆2C-DAS28 > 1.7]; No: 2C-DAS28 non-responders; BMI: body mass index; Sero positive: whether the patient tested positive for anti-CCP or RF antibodies; SJC28: swollen joint count; TJC28: tender joint count; CRP: C-reactive protein levels; normal CRP: CRP < 10 mg/L; PatGA: patient global assessment; PhyGA: physician global assessment; p: p-value from a two-tailed Mann Whitney U test [continuous] or a chi squared test [categorical]).


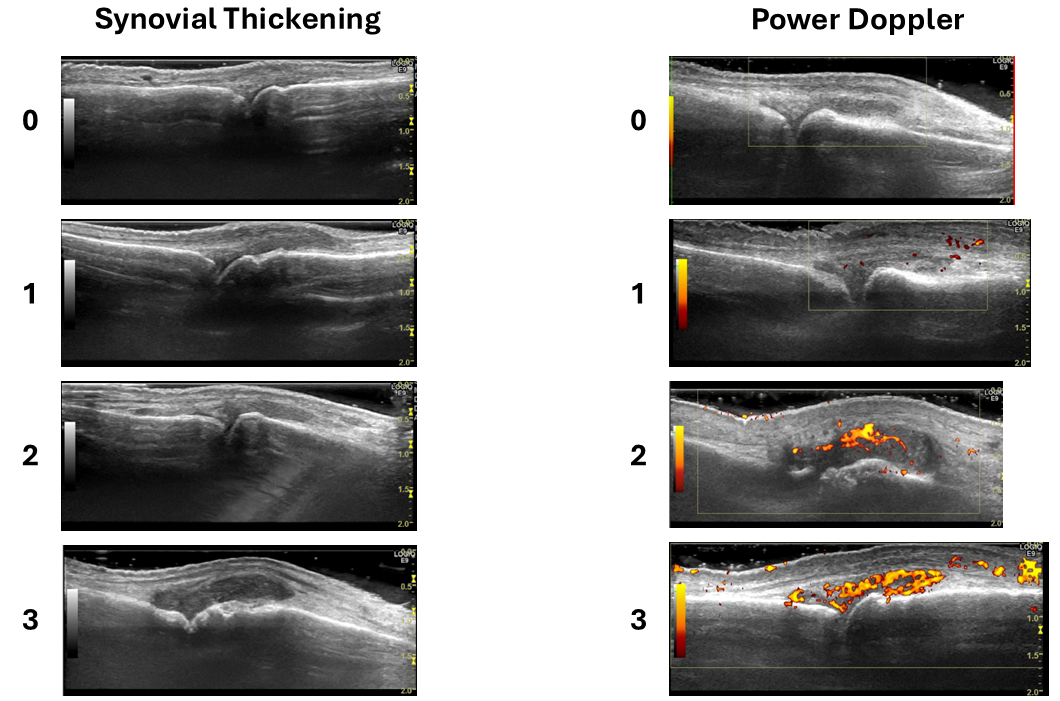


Supplementary Figure S1: Semi-quantitative grading of synovitis in the metacarpophalangeal (MCP) joints according to the EULAR-OMERACT consensus definitions (all images were acquired in the longitudinal dorsal plane using high-frequency transducers).
Left Column (Synovial Thickening): Grayscale images demonstrating synovial hypertrophy from Grade 0 (absence of thickening) to Grade 3 (severe thickening with convexity/bulging of the synovial tissue).
Right Column (Power Doppler): Assessment of active inflammation (vascularity) within the synovium, ranging from Grade 0 (no flow) to Grade 3 (confluent signal involving >50% of the synovial area).

*Alt text Supplementary Figure S1: Two columns of ultrasound images of MCP joints, showing an example image for each semi-quantitative grade of synovial thickening (left) and vascularity (right), in increasing order from top to bottom.*


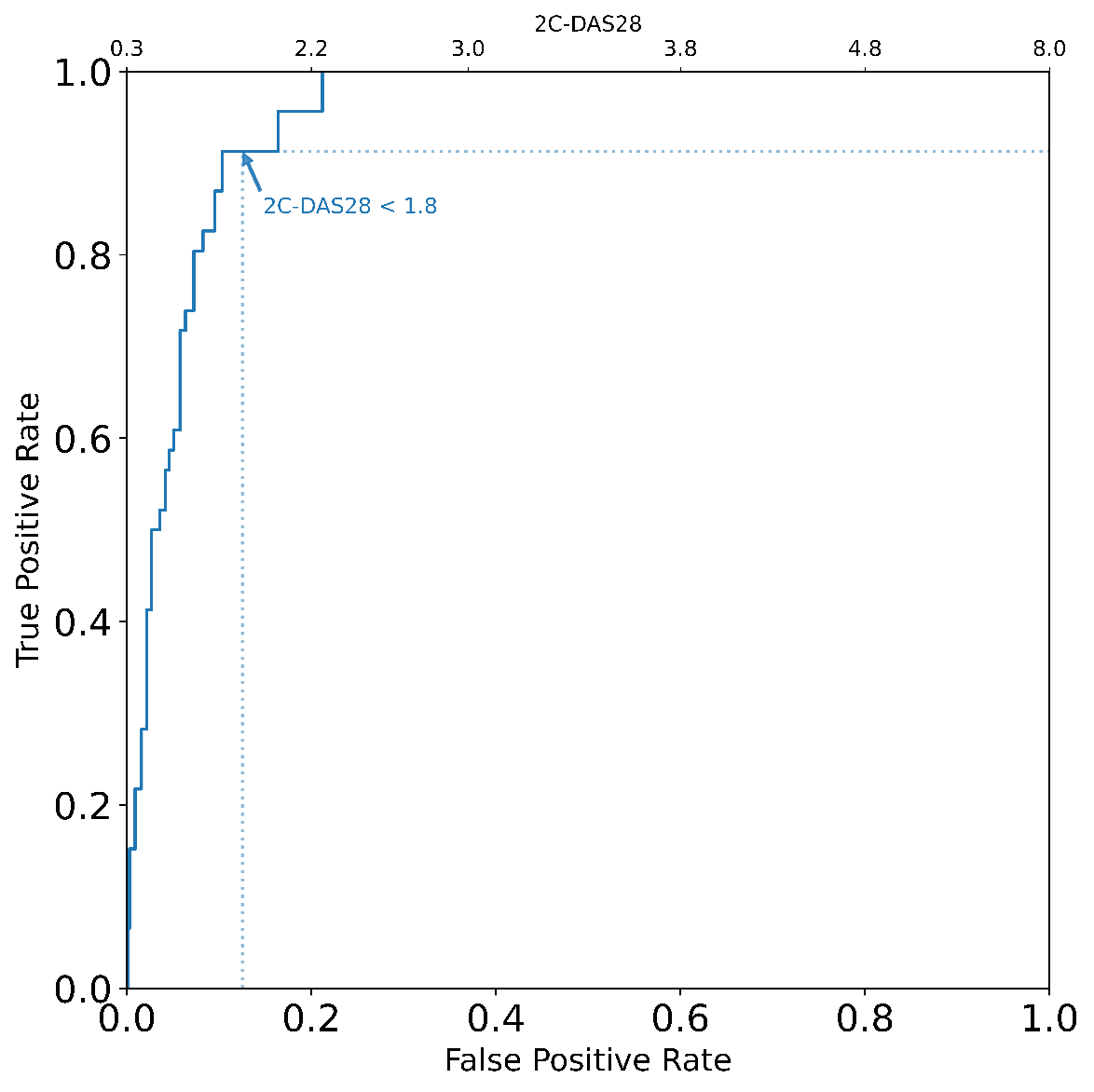


Supplementary Figure S2: ROC curve for thresholds of 2C-DAS28 remission based on Boolean2.0 remission. The ideal threshold was determined using Youden’s J (highlighted).

*Alt text Supplementary Figure S2: ROC curve for varying 2C-DAS28 remission thresholds, showing that the ideal threshold is 2C-DAS28 < 1.8*, with good agreement*.*


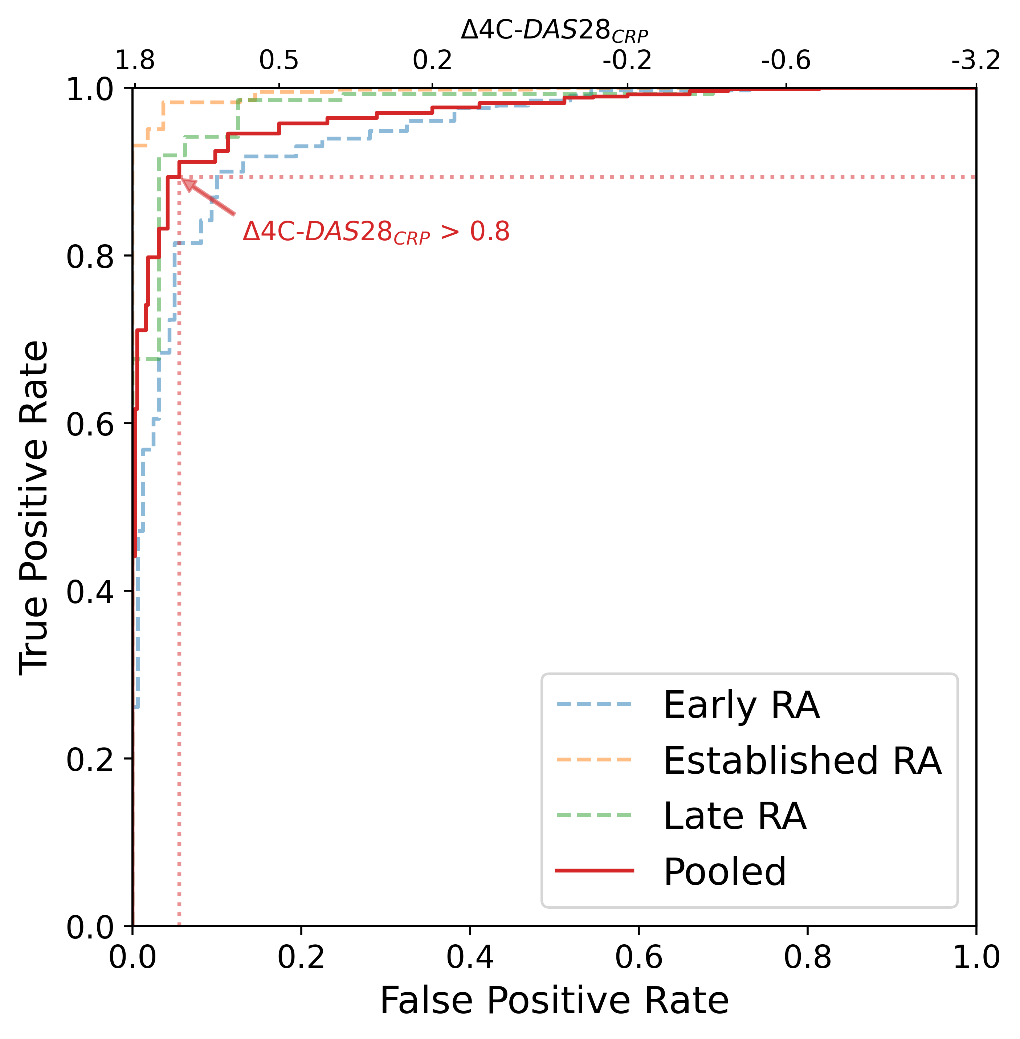


Supplementary Figure S3: ROC curves for moderate change in 4C-DAS28_CRP_, based on the EULAR_ESR_ threshold (Δ4C-DAS_ESR_ > 0.6). The individual curves were pooled using weighted averaging, and Youden’s J was used to identify the ideal threshold (highlighted).

Alt text Supplementary Figure S3: ROC curves for varying change in CRP DAS28 thresholds, showing that the ideal threshold is Δ4C-DAS_CRP_ > 0.8, with good agreement.
